# P-369. Skilled Nursing Facility Characteristics Associated with *Candida auris* Transmission in Maryland

**DOI:** 10.1093/ofid/ofae631.570

**Published:** 2025-01-29

**Authors:** Lauren Smith, Surbhi Leekha, Gary Lin, Jason Falvey, Brittany Grace, Jamie Rubin, Elisabeth Vaeth, Rebecca Perlmutter, Mary-Claire Roghmann

**Affiliations:** University of Maryland School of Medicine, MD; University of Maryland School of Medicine, MD; Johns Hopkins University Applied Physics Laboratory, Baltimore, Maryland; University of Maryland, Baltimore, Maryland; Maryland Department of Health, Baltimore, Maryland; MDH, Baltimore, Maryland; Maryland Department of Health, Baltimore, Maryland, Baltimore, Maryland; Maryland Department of Health, Baltimore, Maryland; University of Maryland School of Medicine, MD

## Abstract

**Background:**

*C. auris* is a multi-drug resistant organism with high morbidity and mortality that is increasing throughout the United States. Skilled nursing facilities (SNFs) play a large role in transmission of this pathogen in healthcare settings. In this study, we compare characteristics of SNFs with and without *C. auris* transmission in Maryland.

Comparison of Resident Characteristics Between Facilities with and without C. auris Transmission

**Figure:**
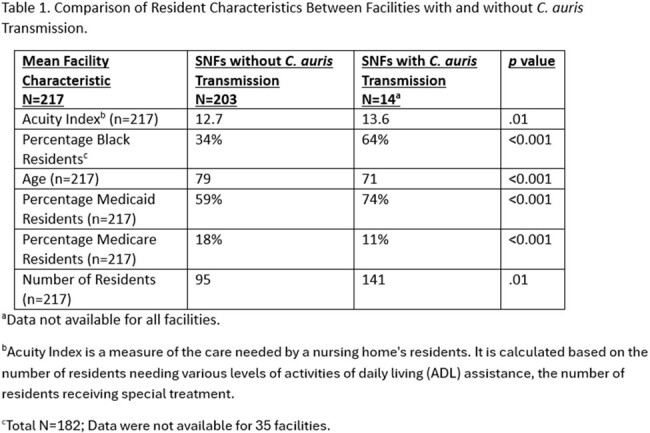

**Methods:**

We conducted a retrospective analysis of all 226 Maryland SNFs to identify characteristics associated with *C. auris* transmission from June 2019, when transmission was first detected in the state, to December 2022. *C. auris* transmission was defined as ≥ 2 cases that were epidemiologically linked to the same SNF. SNF resident and facility characteristics were obtained from the public database Long Term Care Focus (LTCFocus) and the Maryland Health Care Commission (MHCC). A Maryland-specific patient transfer network was built using data derived from Centers for Medicare and Medicaid Services (CMS) fee for service beneficiary claims data linked to the CMS Data Set (Figure 1). Centrality measures were calculated based upon patient transfer networks. We used bivariate analyses to compare facilities with and without *C. auris* transmission.

Comparison of Centrality Measures (Connectedness)Between Facilities with and without C. auris Transmission.

**Figure:**
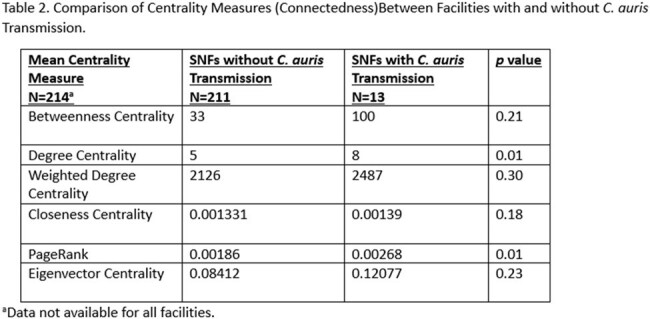

**Results:**

Fifteen SNFs had transmission of C. auris during the study period. SNFs with transmission had a greater number of residents (mean 141 v 95), higher resident acuity, higher percentage of black residents (64% v 34%) and higher percentage of residents on Medicaid (74% v 59%) (Table 1). The patient transfer network of SNFs with transmission generally had higher measures of centrality, including Betweenness Centrality and PageRank (Table 2). Based on the network analysis, facilities that influence and serves as a bridge between other facilities have a higher likelihood of transmission.

Network Characterization of Patient Transfer Network in Maryland Using CMS Data.

**Figure:**
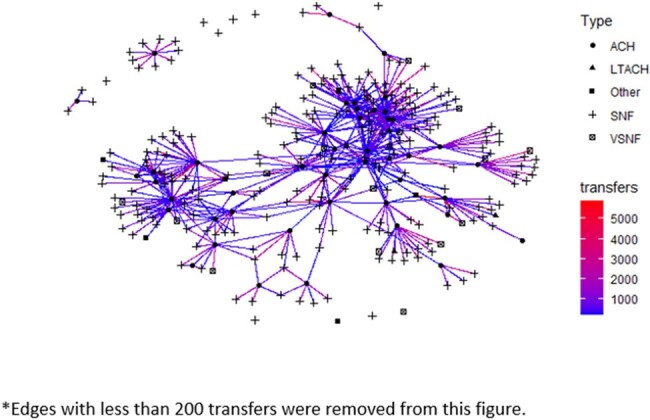

**Conclusion:**

Maryland SNFs with more central patient transfer networks and with a higher proportion of residents on Medicaid, Black residents, and acutely ill were more likely to have *C. auris* transmission. Additional analyses on interactions between facility characteristics and transfer networks are needed to help direct infection prevention efforts.

**Disclosures:**

**All Authors**: No reported disclosures

